# Supplementary material for: Microbiome meta-analysis and cross-disease comparison enabled by the SIAMCAT machine learning toolbox
Source: Genome Biol. 2021 Mar 30;22:93. doi: 10.1186/s13059-021-02306-1 (PMC8008609; doi:10.1186/s13059-021-02306-1)
Supplement: Supplementary file 2 — Additional file 2: Supplementary Tables. Table S1. Information about included datasets. [file 13059_2021_2306_MOESM2_ESM.pdf]

Table S1: Information about included datasets

Metagenomic profiles generated with MetaPhlAn2 and HUMAnN2 were obtained through the curatedMetagenomicsData R package [1]. Profiles created with mOTUs2 or eggNOG4.5 are based on the raw metagenomic data that were preprocessed and profiled in-house (see Methods). Taxonomic profiles generated with the RDP profiler were downloaded from [2]. For some datasets, the raw metagenomic sequencing data were not deposited on SRA or ENA, but they were included in the meta-analysis of [2], as the original authors had provided the data to Duvallet *et al.* upon request.

Dataset ID	Ref.	Source of raw metagenomic data	Classification task	Disease abbr.	Profiler	Mean (sd) sequencing depth
Alkanani 2015	[3]		Type 1 diabetes	T1D	RDP	
Baxter 2016	[4]	ENA accession: PRJNA290926	Colorectal cancer	CRC	RDP	61,795.42 (±47,935.26)
Bedarf 2017	[5]	ENA accession: ERP019674	Parkinson's disease	PAR	mOTUs_v2 eggNOG 4.5	31,907,463 (±10,583,224)
Chen 2012	[6]	ENA accession: SRP009633	Colorectal cancer	CRC	RDP	
Dinh 2015	[7]	ENA accession: PRJNA233597	HIV infection	HIV	RDP	10,194.25 (±4,505.60)
Feng 2015	[8]	ENA accession: ERP008729	Colorectal cancer	CRC	MetaPhlAn2 HUMAnN2 mOTUs_v2 eggNOG 4.5	27,326,375 (±4,306,354)
			Advanced adenoma	ADA		
Franzosa 2019	[9]	ENA accession: PRJNA400072	Crohn's disease	CD	mOTUs_v2 eggNOG 4.5	21,426,878 (±16,657,149)
			Ulcerative colitis	UC		
Gevers 2014	[10]	ENA accession: PRJNA237362	Inflammatory bowel disease	IBD	RDP	63,584.85 (±74,568.79)
Goodrich 2014	[11]	ENA accession: PRJEB6702 and PRJEB6705	Obesity	OB	RDP	
Hannigan 2017	[12]	ENA accession: PRJNA389927	Colorectal cancer	CRC	MetaPhlAn2 HUMAnN2 MetaPhlAn2 HUMAnN2	2,930,666 (±2,459,779)
			Advanced adenoma	ADA		

He 2017	[13]	ENA accession: PRJEB15371	Crohn’s disease	CD	mOTUs_v2 eggNOG 4.5	27,685,675 (±3,815,790)
Hoyles 2018	[14]	ENA accession: PRJEB14215	Non-alcoholic fatty liver disease	NAFLD	mOTUs_v2 eggNOG 4.5	20,121,522 (±5,766,812)
Jie 2017	[15]	ENA accession: ERP023788	Atherosclerotic cardiovascular disease	ACVD	mOTUs_v2 eggNOG 4.5	27,014,710 (±5,447,881)
Kang 2013	[16]	ENA accession: PRJNA168470	Autism spectrum disorder	ASD	RDP	53,647.45 (±7,844.45)
Karlsson 2013	[17]	ENA accession: PRJEB1786	Type 2 diabetes	T2D	MetaPhlAn2 HUMAnN2 mOTUs_v2 eggNOG 4.5	15,496,907 (±8,813,177)
			Impaired glucose tolerance	IGT		
Kushugolova 2018	[18]	ENA accession: PRJEB17632	Metabolic syndrome	MS	mOTUs_v2 eggNOG 4.5	34,480,585 (±9,797,315)
Le Chatelier 2013	[19]	ENA accession: PRJEB4336	Obesity	OB	mOTUs_v2 eggNOG 4.5	34,479,440 (±11,613,906)
Lewis 2015	[20]	ENA accession: SRP057027	Crohn’s disease	CD	mOTUs_v2 eggNOG 4.5	11,527,575 (±9,075,748)
Li 2017	[21]	ENA accession: PRJEB13870	Hypertension	HTN	MetaPhlAn2 HUMAnN2 mOTUs_v2 eggNOG 4.5	22,415,253 (±2,636,717)
			Pre hypertension	pHTN		
Lloyd-Price 2019	[22]	ENA accession: PRJNA398089	Croh’s disease	CD	mOTUs_v2 eggNOG 4.5	12,842,381 (±5,011,535)
			Ulcerative colitis	UC		
Loomba 2017	[23]	ENA accession: PRJNA373901	Non-alcoholic fatty liver disease	NAFLD	mOTUs_v2 eggNOG 4.5	28,850,336 (±19,049,180)
Lozupone 2013	[24]	ENA accession: PRJEB4335	HIV infection	HIV	RDP	21,681.9 (±15,592.95)
Morgan 2012	[25]	ENA accesion: PRJNA82111	Inflammatory bowel disease	IBD	RDP	4,928.39 (±2,522.36)
Nielsen 2014	[26]	ENA accession:	Inflammatory	IBD	MetaPhlAn2	28,387,517

		PRJEB1220	bowel disease		HUMAnN2	(±9,902,539)
			Crohn’s disease	CD	mOTUs_v2<br>eggNOG 4.5	
			Ulcerative colitis	UC		
Noguera-Julian 2016	[27]	ENA accession: PRJNA307231	HIV infection	HIV	RDP	210,881.8 (±162,942.9)
Papa 2012	[28]		Inflammatory bowel disease	IBD	RDP	
Qin 2012	[29]	ENA accession: PRJNA422434	Type 2 diabetes	T2D	MetaPhlAn2<br>HUMAnN2<br>mOTUs_v2<br>eggNOG 4.5	20,387,391 (±6,265,854)
Qin 2014	[30]	ENA accession: ERP005860	Liver disease	LIV	MetaPhlAn2<br>HUMAnN2<br>mOTUs_v2<br>eggNOG 4.5	19,608,423 (±16,248,984)
Ross 2015	[31]	ENA accession: PRJNA273824	Obesity	OB	RDP	15,176.92 (±8,374.48)
Scheperjans 2015	[32]	ENA accession: PRJEB4927	Parkinson’s disease	PAR	RDP	17,224.44 (±3,225.63)
Scher 2013	[33]	ENA accession: PRJNA203810	Arthritis	ART	RDP	13,924.89 (±8,868.68)
Schubert 2014	[34]		*Clostridioides difficile* infection	CDI	RDP	
			Diarrhea without *Clostridioides difficile* infection	nCDI		
Singh 2015	[35]	http://dx.doi.org/10.6084/m9.figshare.1447256	Enteric diarrheal disease	EDD	RDP	
Son 2015	[36]	ENA accession: PRJNA282013	Autism spectrum disorder	ASD	RDP	641,985.3 (±133,461.7)
Thomas 2019a	[37]	ENA accession: PRJNA447983	Colorectal cancer	CRC	MetaPhlAn2<br>HUMAnN2<br>mOTUs_v2<br>eggNOG 4.5	22,492,399 (±12,201,510)
			Advanced adenoma	ADA		
Thomas 2019b			Colorectal cancer	CRC		
Turnbaugh 2009	[38]	https://gordonlab.wustl.edu/NatureTwins_2008/Turn	Obesity	OB	RDP	

		baughNature_11_30_08.html				
Vincent 2013	[39]		*Clostridioides difficile* infection	CDI	RDP	
Vogtmann 2016	[40]	ENA accession: PRJEB12449	Colorectal cancer	CRC	MetaPhlAn2<br>HUMAnN2<br>mOTUs_v2<br>eggNOG 4.5	35,168,861 (±7,888,187)
Wang 2012	[41]	ENA accession: SRP005150	Colorectal cancer	CRC	RDP	
Wen 2017	[42]	ENA accession: SRP100575	Ankylosing spondylitis	AS	mOTUs_v2<br>eggNOG 4.5	18,948,792 (±8,463,097)
Willing 2019	[43]		Inflammatory bowel disease	IBD	RDP	
Wirbel 2019	[44]	ENA accession: PRJEB27928	Colorectal cancer	CRC	mOTUs_v2<br>eggNOG 4.5	23,266,126 (±10,596,669)
Wong 2013	[45]	ENA accession: SRP011160	Non-alcoholic steatohepatitis	NASH	RDP	7,931.5 (±1,626.39)
Yachida 2019	[46]	ENA accession: PRJDB4176	Colorectal cancer	CRC	mOTUs_v2<br>eggNOG 4.5	23,135,878 (±6,651,418)
			Advanced adenoma	ADA		
Yu 2015	[47]	ENA accession: PRJEB10878	Colorectal cancer	CRC	MetaPhlAn2<br>HUMAnN2<br>mOTUs_v2<br>eggNOG 4.5	28,158,833 (±4,978,013)
Zeller 2014	[48]	ENA accession: ERP005534	Colorectal cancer	CRC	MetaPhlAn2<br>HUMAnN2<br>mOTUs_v2<br>eggNOG 4.5<br>RDP	WGS: 34,463,015 (±14,101,235)<br>Amplicon: 277,964 (±194,631)
			Advanced adenoma	ADA		
Zhang 2013	[49]	ENA accession: PRJNA174838	Liver disease	LIV	RDP	4,707.18 (±3,195.73)
Zhang 2015	[50]	ENA accession: PRJEB6997	Rheumatoid arthiris	RA	mOTUs_v2<br>eggNOG 4.5	27.271.221 (±10.057.577)

Zhu 2013	[51]	MG-RAST, study mgp1195	Non-alcoholic steatohepatitis	NASH	RDP	
Zupancic 2012	[52]	ENA accession: PRJNA46319	Obesity	OB	RDP	501,023.4 (±522,488)

1. Pasolli E, Schiffer L, Manghi P, Renson A, Obenchain V, Truong DT, et al. Accessible, curated metagenomic data through ExperimentHub. Nat Methods. 2017;14: 1023–1024.

2. Duvallet C, Gibbons SM, Gurry T, Irizarry RA, Alm EJ. Meta-analysis of gut microbiome studies identifies disease-specific and shared responses. Nat Commun. 2017;8: 1784.

3. Alkanani AK, Hara N, Gottlieb PA, Ir D, Robertson CE, Wagner BD, et al. Alterations in Intestinal Microbiota Correlate With Susceptibility to Type 1 Diabetes. Diabetes. 2015;64: 3510–3520.

4. Baxter NT, Ruffin MT 4th, Rogers MAM, Schloss PD. Microbiota-based model improves the sensitivity of fecal immunochemical test for detecting colonic lesions. Genome Med. 2016;8: 37.

5. Bedarf JR, Hildebrand F, Coelho LP, Sunagawa S, Bahram M, Goeser F, et al. Functional implications of microbial and viral gut metagenome changes in early stage L-DOPA-naïve Parkinson's disease patients. Genome Med. 2017;9: 39.

6. Chen W, Liu F, Ling Z, Tong X, Xiang C. Human intestinal lumen and mucosa-associated microbiota in patients with colorectal cancer. PLoS One. 2012;7: e39743.

7. Dinh DM, Volpe GE, Duffalo C, Bhalchandra S, Tai AK, Kane AV, et al. Intestinal microbiota, microbial translocation, and systemic inflammation in chronic HIV infection. J Infect Dis. 2015;211: 19–27.

8. Feng Q, Liang S, Jia H, Stadlmayr A, Tang L, Lan Z, et al. Gut microbiome development along the colorectal adenoma–carcinoma sequence. Nat Commun. 2015;6: 6528.

9. Franzosa EA, Sirota-Madi A, Avila-Pacheco J, Fornelos N, Haiser HJ, Reinker S, et al. Gut microbiome structure and metabolic activity in inflammatory bowel disease. Nat Microbiol. 2019;4: 293–305.

10. Gevers D, Kugathasan S, Denson LA. The treatment-naive microbiome in new-onset Crohn's disease. Cell Host Microbe. 2014. Available: https://www.sciencedirect.com/science/article/pii/S1931312814000638

11. Goodrich JK, Waters JL, Poole AC, Sutter JL, Koren O, Blekhman R, et al. Human genetics shape the gut microbiome. Cell. 2014;159: 789–799.

12. Hannigan GD, Duhaime MB, Ruffin MT 4th, Koumpouras CC, Schloss PD. Diagnostic Potential and Interactive Dynamics of the Colorectal Cancer Virome. MBio. 2018;9. doi:10.1128/mBio.02248-18

13. He Q, Gao Y, Jie Z, Yu X, Laursen JM, Xiao L, et al. Two distinct metacommunities characterize the gut microbiota in Crohn's disease patients. Gigascience. 2017;6: 1–11.

14. Hoyles L, Fernández-Real J-M, Federici M, Serino M, Abbott J, Charpentier J, et al. Molecular phenomics and metagenomics of hepatic steatosis in non-diabetic obese women. Nat Med. 2018;24: 1070–1080.

15. Jie Z, Xia H, Zhong S-L, Feng Q, Li S, Liang S, et al. The gut microbiome in atherosclerotic cardiovascular disease. Nat Commun. 2017;8: 845.

16. Kang D-W, Park JG, Ilhan ZE, Wallstrom G, Labaer J, Adams JB, et al. Reduced incidence of Prevotella and other fermenters in intestinal microflora of autistic children. PLoS One. 2013;8: e68322.

17. Karlsson FH, Tremaroli V, Nookaew I, Bergström G, Behre CJ, Fagerberg B, et al. Gut metagenome in European women with normal, impaired and diabetic glucose control. Nature. 2013;498: 99–103.

18. Kushugulova A, Forslund SK, Costea PI, Kozhakhmetov S, Khassenbekova Z, Urazova M, et al. Metagenomic analysis of gut microbial communities from a Central Asian population. BMJ Open. 2018;8: e021682.

19. Le Chatelier E, Nielsen T, Qin J, Prifti E, Hildebrand F, Falony G, et al. Richness of human gut microbiome correlates with metabolic markers. Nature. 2013;500: 541–546.

20. Lewis JD, Chen EZ, Baldassano RN, Otley AR, Griffiths AM, Lee D, et al. Inflammation, Antibiotics, and Diet as Environmental Stressors of the Gut Microbiome in Pediatric Crohn's Disease. Cell Host Microbe. 2015;18: 489–500.

21. Li J, Zhao F, Wang Y, Chen J, Tao J, Tian G, et al. Gut microbiota dysbiosis contributes to the development of hypertension. Microbiome. 2017;5: 14.

22. Lloyd-Price J, Arze C, Ananthakrishnan AN, Schirmer M, Avila-Pacheco J, Poon TW, et al. Multi-omics of the gut microbial ecosystem in inflammatory bowel diseases. Nature. 2019;569: 655–662.

23. Loomba R, Seguritan V, Li W, Long T, Klitgord N, Bhatt A, et al. Gut Microbiome-Based Metagenomic Signature for Non-invasive Detection of Advanced Fibrosis in Human Nonalcoholic Fatty Liver Disease. Cell Metab. 2017;25: 1054–1062.e5.

24. Lozupone CA, Li M, Campbell TB, Flores SC, Linderman D, Gebert MJ, et al. Alterations in the gut microbiota associated with HIV-1 infection. Cell Host Microbe. 2013;14: 329–339.

25. Morgan XC, Tickle TL, Sokol H, Gevers D, Devaney KL, Ward DV, et al. Dysfunction of the intestinal microbiome in inflammatory bowel disease and treatment. Genome Biol. 2012;13: R79.

26. Nielsen HB, Almeida M, Juncker AS, Rasmussen S, Li J, Sunagawa S, et al. Identification and assembly of genomes and genetic elements in complex metagenomic samples without using reference genomes. Nat Biotechnol. 2014;32: 822–828.

27. Noguera-Julian M, Rocafort M, Guillén Y, Rivera J, Casadellà M, Nowak P, et al. Gut Microbiota Linked to Sexual Preference and HIV Infection. EBioMedicine. 2016;5: 135–146.

28. Papa E, Docktor M, Smillie C, Weber S, Preheim SP, Gevers D, et al. Non-invasive

mapping of the gastrointestinal microbiota identifies children with inflammatory bowel disease. PLoS One. 2012;7: e39242.

29. Qin J, Li Y, Cai Z, Li S, Zhu J, Zhang F, et al. A metagenome-wide association study of gut microbiota in type 2 diabetes. Nature. 2012;490: 55–60.

30. Qin N, Yang F, Li A, Prifti E, Chen Y, Shao L, et al. Alterations of the human gut microbiome in liver cirrhosis. Nature. 2014;513: 59–64.

31. Ross MC, Muzny DM, McCormick JB, Gibbs RA, Fisher-Hoch SP, Petrosino JF. 16S gut community of the Cameron County Hispanic Cohort. Microbiome. 2015;3: 7.

32. Scheperjans F, Aho V, Pereira PAB, Koskinen K, Paulin L, Pekkonen E, et al. Gut microbiota are related to Parkinson's disease and clinical phenotype. Mov Disord. 2015;30: 350–358.

33. Scher JU, Sczesnak A, Longman RS, Segata N, Ubeda C, Bielski C, et al. Expansion of intestinal Prevotella copri correlates with enhanced susceptibility to arthritis. Elife. 2013;2: e01202.

34. Schubert AM, Rogers MAM, Ring C, Mogle J, Petrosino JP, Young VB, et al. Microbiome data distinguish patients with Clostridium difficile infection and non-C. difficile-associated diarrhea from healthy controls. MBio. 2014;5: e01021–14.

35. Singh P, Teal TK, Marsh TL, Tiedje JM, Mosci R, Jernigan K, et al. Intestinal microbial communities associated with acute enteric infections and disease recovery. Microbiome. 2015;3: 45.

36. Son JS, Zheng LJ, Rowehl LM, Tian X, Zhang Y, Zhu W, et al. Comparison of Fecal Microbiota in Children with Autism Spectrum Disorders and Neurotypical Siblings in the Simons Simplex Collection. PLoS One. 2015;10: e0137725.

37. Thomas AM, Manghi P, Asnicar F, Pasolli E, Armanini F, Zolfo M, et al. Metagenomic analysis of colorectal cancer datasets identifies cross-cohort microbial diagnostic signatures and a link with choline degradation. Nat Med. 2019;25: 667–678.

38. Turnbaugh PJ, Hamady M, Yatsunenko T, Cantarel BL, Duncan A, Ley RE, et al. A core gut microbiome in obese and lean twins. Nature. 2009;457: 480–484.

39. Vincent C, Stephens DA, Loo VG, Edens TJ, Behr MA, Dewar K, et al. Reductions in intestinal Clostridiales precede the development of nosocomial Clostridium difficile infection. Microbiome. 2013;1: 18.

40. Vogtmann E, Hua X, Zeller G, Sunagawa S, Voigt AY, Hercog R, et al. Colorectal Cancer and the Human Gut Microbiome: Reproducibility with Whole-Genome Shotgun Sequencing. PLoS One. 2016;11: e0155362.

41. Wang T, Cai G, Qiu Y, Fei N, Zhang M, Pang X, et al. Structural segregation of gut microbiota between colorectal cancer patients and healthy volunteers. ISME J. 2012;6: 320–329.

42. Wen C, Zheng Z, Shao T, Liu L, Xie Z, Le Chatelier E, et al. Quantitative metagenomics reveals unique gut microbiome biomarkers in ankylosing spondylitis. Genome Biol. 2017;18: 142.

43. Willing BP, Dicksved J, Halfvarson J, Andersson AF, Lucio M, Zheng Z, et al. A pyrosequencing study in twins shows that gastrointestinal microbial profiles vary with inflammatory bowel disease phenotypes. Gastroenterology. 2010;139: 1844–1854.e1.

44. Wirbel J, Pyl PT, Kartal E, Zych K, Kashani A, Milanese A, et al. Meta-analysis of fecal metagenomes reveals global microbial signatures that are specific for colorectal cancer. Nat Med. 2019;25: 679–689.

45. Wong VW-S, Tse C-H, Lam TT-Y, Wong GL-H, Chim AM-L, Chu WC-W, et al. Molecular characterization of the fecal microbiota in patients with nonalcoholic steatohepatitis--a longitudinal study. PLoS One. 2013;8: e62885.

46. Yachida S, Mizutani S, Shiroma H, Shiba S, Nakajima T, Sakamoto T, et al. Metagenomic and metabolomic analyses reveal distinct stage-specific phenotypes of the gut microbiota in colorectal cancer. Nat Med. 2019;25: 968–976.

47. Yu J, Feng Q, Wong SH, Zhang D, Liang QY, Qin Y, et al. Metagenomic analysis of faecal microbiome as a tool towards targeted non-invasive biomarkers for colorectal cancer. Gut. 2017;66: 70–78.

48. Zeller G, Tap J, Voigt AY, Sunagawa S, Kultima JR, Costea PI, et al. Potential of fecal microbiota for early-stage detection of colorectal cancer. Mol Syst Biol. 2014;10. Available: https://www.embopress.org/doi/abs/10.15252/msb.20145645

49. Zhang Z, Zhai H, Geng J, Yu R, Ren H, Fan H, et al. Large-scale survey of gut microbiota associated with MHE Via 16S rRNA-based pyrosequencing. Am J Gastroenterol. 2013;108: 1601–1611.

50. Zhang X, Zhang D, Jia H, Feng Q, Wang D, Liang D, et al. The oral and gut microbiomes are perturbed in rheumatoid arthritis and partly normalized after treatment. Nat Med. 2015;21: 895–905.

51. Zhu L, Baker SS, Gill C, Liu W, Alkhouri R, Baker RD, et al. Characterization of gut microbiomes in nonalcoholic steatohepatitis (NASH) patients: a connection between endogenous alcohol and NASH. Hepatology. 2013;57: 601–609.

52. Zupancic ML, Cantarel BL, Liu Z, Drabek EF, Ryan KA, Cirimotich S, et al. Analysis of the gut microbiota in the old order Amish and its relation to the metabolic syndrome. PLoS One. 2012;7: e43052.
